# Anaphylactic Reactions to COVID-19 Vaccines: An Updated Assessment Based on Pharmacovigilance Data

**DOI:** 10.3390/vaccines11030613

**Published:** 2023-03-08

**Authors:** Fotini Boufidou, Sophia Hatziantoniou, Kalliopi Theodoridou, Helena C. Maltezou, Konstantinos Vasileiou, Cleo Anastassopoulou, Snežana Medić, Athanasios Tsakris

**Affiliations:** 1 Neurochemistry and Biological Markers Unit, 1st Department of Neurology, Eginition Hospital, School of Medicine, National and Kapodistrian University of Athens, 11528 Athens, Greece; 2 Laboratory of Pharmaceutical Technology, Department of Pharmacy, School of Health Sciences, University of Patras, 26504 Patras, Greece; 3 Department of Microbiology, Medical School, National and Kapodistrian University of Athens, 11527 Athens, Greece; 4 Department of Microbiology, Andreas Syggros Hospital for Skin and Venereal Diseases, National and Kapodistrian University of Athens, 15772 Athens, Greece; 5 Directorate of Research, Studies and Documentation, National Public Health Organization, 15123 Athens, Greece; 6 Department of Pharmacy, School of Health Sciences, University of Patras, 26504 Patras, Greece; 7 Department of Epidemiology, Faculty of Medicine, University of Novi Sad, 21000 Novi Sad, Serbia; 8 Center for Disease Control and Prevention, Institute of Public Health of Vojvodina, 21000 Novi Sad, Serbia

**Keywords:** COVID-19 vaccines, allergic reactions, anaphylaxis, hypersensitivity, polyethylene glycol (PEG), polysorbates

## Abstract

This study aimed at producing an updated assessment of the incidence of anaphylaxis associated with COVID-19 vaccines based on pharmacovigilance data. Anaphylactic reaction and anaphylactic shock data post-COVID-19-vaccination reported from week 52, 2020 to week 1 or week 2, 2023 were collected from the VAERS and EudraVigilance databases, respectively, and analyzed comparatively. Incidence rates were calculated using the corresponding administered vaccine doses as denominators for all licensed vaccines and both platform types (mRNA or vectored). The latest data from the present analysis showed lower anaphylaxis incidence associated with COVID-19 vaccination compared to previous estimates from week 52, 2020 to week 39, 2021 (anaphylactic reaction: 8.96 (95% CI 8.80–9.11)/million doses overall (EEA: 14.19 (95% CI 13.92–14.47)/million/US: 3.17 (95% CI 3.03–3.31)/million); anaphylactic shock: 1.46 (95% CI 1.39–1.52)/million doses overall (EEA: 2.47 (95% CI 2.36–2.58)/million/US: 0.33 (95% CI 0.29–0.38)/million)). Incidence rates varied by vaccine and were higher as captured in EudraVigilance compared to the VAERS and for vectored compared to mRNA vaccines. Most reported cases had a favorable outcome. The extremely rare fatalities (overall rates across continents 0.04 (95% CI 0.03–0.06)/million doses for anaphylactic reaction and 0.02 (95% CI 0.01–0.03)/million vaccine doses for anaphylactic shock) were also associated with vector-rather than mRNA-based vaccines. The diminished incidence of anaphylaxis post-vaccination with COVID-19 vaccines offers assurance about their safety, as does the continuous potential adverse events monitoring through specialized pharmacovigilance databases.

## 1. Introduction

Coronavirus disease 2019 (COVID-19) vaccines, together with non-pharmaceutical interventions (face masks and social distancing), form the key pillars of our armamentarium against the devastating public health and socioeconomic consequences of the pandemic. Ironically, the amazing scientific accomplishment of the swift development and approval of vaccines for emergency use, just over a year after the emergence of severe acute respiratory syndrome coronavirus 2 (SARS-CoV-2), raised concerns about their safety among the public, despite the excess mortality associated with the pandemic [1]. Skepticism was also heightened by the use of novel vaccine platforms, as in the case of the never-before-used-at-such-scale mRNA-based vaccines, as well as by mistrust in authorities in some countries, which resulted in a lack of the confidence in vaccines and affected vaccine uptake [2,3].

The use of any vaccine (or systemic medication) is potentially associated with a risk of adverse events, including allergic reactions; however, severe cases and fatal outcomes are extremely rare post-vaccination [4,5]. Following deployment of the first COVID-19 vaccines, reports of anaphylactic reactions, presumably due to the polyethylene glycol contained in the novel mRNA vaccines, received significant attention in the press and social media [6,7]. Although rare, anaphylaxis may be life-threatening as multiple organs of different systems may be acutely affected, including the cutaneous, gastrointestinal, respiratory, and cardiovascular systems [4]. The underlying mechanisms can be immunological, non-immunological (previously termed anaphylactoid reactions), or idiopathic, with all three types leading to a similar clinical picture [4].

Anaphylactic reactions are categorized as adverse events of special interest (AESI) due to their potential for altering the risk–benefit profile of medicines and vaccines [4]. Their significant medical and scientific merit and use necessitate ongoing monitoring, communication with the vaccine industry, healthcare professionals, and the public, and immediate medical action by regulators. Hence, reporting anaphylactic reactions to pharmacovigilance systems is critical for the characterization of the safety profile of vaccines. In Europe, suspected adverse reactions to medicines, including vaccines, are monitored and analyzed by EudraVigilance, which is managed by the European Medicine Agency (ΕΜA) [8]. In the United States (US), possible adverse events post-vaccination are recorded and analyzed by the Vaccine Adverse Event Reporting System (VAERS), an early-warning passive surveillance system supervised by the US Centers for Disease Control and Prevention (CDC) and the Food and Drug Administration (FDA) [9]. Both programs are passive reporting systems where healthcare providers, vaccine manufacturers, and the public can report adverse events. As such, both systems are subject to misreporting biases, the potential impact of which is nonetheless minimized by internal evaluation of reported adverse events [8,9,10].

Recently we demonstrated that anaphylaxis rates associated with COVID-19 vaccines, estimated at 10.67 cases/million vaccine doses (range: 7.99–19.39 cases/million doses depending on the vaccine), are comparable to those of traditional vaccines [11]. We also comparatively assessed the incidence of the most commonly reported allergic reactions post-COVID-19-vaccination with licensed vaccines in Europe and the US from week 52, 2020 to week 39, 2021 [12]. The need for vaccination against the ever-changing SARS-CoV-2 variants, given our waning infection-, vaccine-induced, or hybrid immunity, may continue. The aim of the present work was to produce an updated assessment of anaphylactic reactions to COVID-19 vaccines based on the available real-life pharmacovigilance data.

## 2. Materials and Methods

### 2.1. Pharmacovigilance Data Retrieval

Data from anaphylactic reactions and anaphylactic shock cases following COVID-19 vaccination from week 52, 2020 (ending on 31 December 2020) to week 2 or week 1, 2023 (ending on 13 January or 6 January 2023, respectively) were collected from EudraVigilance for the European Economic Area (EEA) and from the VAERS for the US [8,9]. We collected the available anaphylaxis data from EudraVigilance by age and sex, selecting “anaphylactic reaction” and “anaphylactic shock” reaction groups that are listed under “immune system disorders”. The same grouping (by age and sex) was selected on the VAERS database, and the data were mined by selecting either symptom. We also selected “all events” and “death” from the “other characteristics” selection list. The data on “life threatening” and “permanent disability” were combined and reported as “complicated course”. The data source for the VAERS was the public use database where data are coded using the MedDRA system. Typically, the Brighton Collaboration criteria regarding anaphylaxis are applied only in cases for which additional follow-up is conducted by CDC and FDA investigators (VAERS). Data were collected and analyzed for all licensed vaccines, which included mRNA-1273 (Moderna), BNT162b2 (Pfizer-BioNTech), ChAdOx1-S (Oxford/AstraZeneca), and AD26.COV2.S (Janssen/Johnson & Johnson). ChAdOx1-S is not licensed in the US.

### 2.2. Administered Vaccine Doses

The total numbers of administered COVID-19 vaccine doses as of 13 January or 6 January 2023 were retrieved from the European Centre for Disease Prevention and Control for the EEA and from the Centers for Disease Control and Prevention for the US [13,14]. The EEA includes 27 European Union (EU) countries (Austria, Belgium, Bulgaria, Croatia, Cyprus, Czechia, Denmark, Estonia, Finland, France, Germany, Greece, Hungary, Ireland, Italy, Latvia, Lithuania, Luxembourg, Malta, Netherlands, Poland, Portugal, Romania, Slovakia, Slovenia, Spain, Sweden), as well as Iceland, Liechtenstein, and Norway.

### 2.3. Estimation of Anaphylaxis Incidence

To estimate anaphylaxis incidence, the reported numbers of anaphylactic reactions and anaphylactic shock cases were expressed per million administered doses for each licensed vaccine. Overall incidence rates were calculated by summing the reported anaphylaxis cases for all vaccines and dividing by the corresponding total number of vaccine doses administered during the study period [13,14].

### 2.4. Statistical Analysis

Data were analyzed using the SPSS statistical program (version 28; IBM, NY, USA). Incidence rates (IR) and the corresponding 95% confidence intervals (CIs) of reported cases and fatalities were calculated per million vaccine doses. Statistical differences in the anaphylactic reaction and anaphylactic shock reporting rates between the two pharmacovigilance systems (EudraVigilance and VAERS) and technological platforms of vaccines (mRNA and adenovirus vectored) were assessed by Pearson’s chi-squared asymptotic significance (*p*) value. Odds ratios (OR), including 95% CIs, were estimated for all comparisons.

## 3. Results

### 3.1. Incidence of Anaphylactic Reaction and Anaphylactic Shock Cases

As of 13 January or 6 January 2023, a total of 12,549 cases of anaphylactic reactions post-COVID-19-vaccination, i.e., 10,439 and 2110 cases, were registered in EudraVigilance and the VAERS, respectively. The distribution and description of anaphylactic reaction cases by vaccine are shown in Table 1 and Table 2, respectively. Anaphylactic shock cases post-COVID-19-vaccination recorded in EudraVigilance and the VAERS amounted to 1817 and 222, respectively, for a total of 2039 cases; their description and distribution by vaccine manufacturer are shown in Table 3 and Table 4, respectively. Administered vaccine doses for licensed vaccines for which the manufacturer was known totaled 735,458,789 in the EEA and 665,648,309 in the US, combining to give a total of 1,401,107,098 doses of vaccine. Thus, the overall incidence of anaphylactic reactions was 8.96 (95% CI 8.80–9.11)/million doses (14.19 (95% CI 13.92–14.47)/million in the EEA and 3.17 (95% CI 3.03–3.31)/million in the US) and of anaphylactic shock 1.46 ((95% CI 1.39–1.52)/million vaccine doses (2.47 (95% CI 2.36–2.58)/million in the EEA and 0.33 (95% CI 0.29–0.38)/million in the US).

### 3.2. Anaphylaxis Incidence Variation by Vaccine

**Figure 1:**
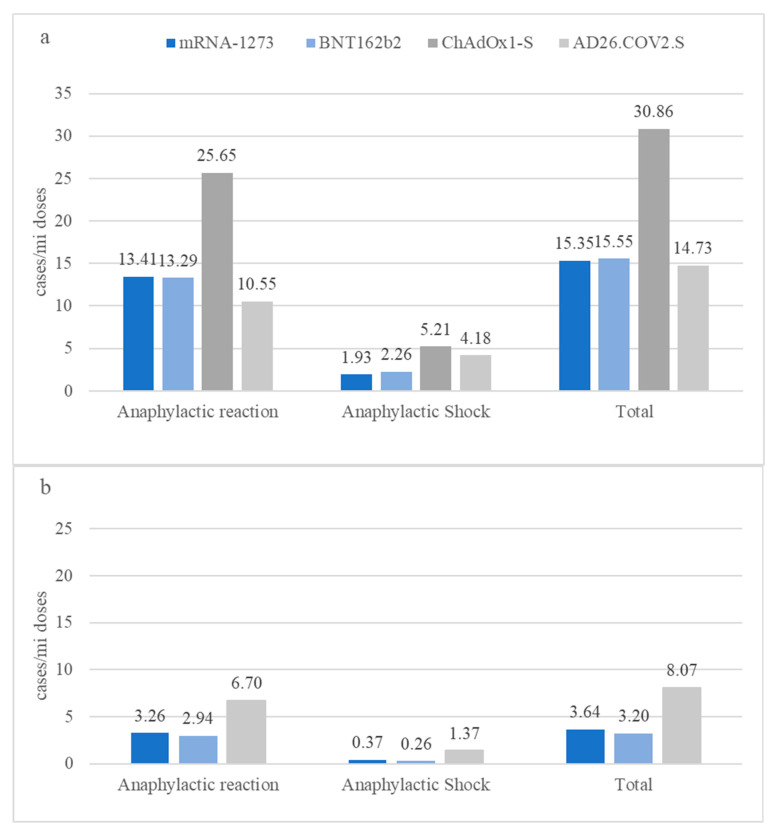
Rates of estimated anaphylactic reaction and anaphylactic shock post-COVID-19-vaccination for licensed vaccines, as reported by the EudraVigilance (**a**) and VAERS (**b**) databases, expressed per million administered doses from week 52, 2020 (ending on 31 December 2020) to week 2 (**a**) or week 1 (**b**), 2023 (ending on 13 January or 6 January 2023, respectively).

The incidence of anaphylactic reactions following COVID-19 vaccination reported by EudraVigilance varied considerably by vaccine, ranging from a minimum of 10.55 (95% CI 8.96–12.14)/million doses for AD26.COV2.S to a maximum of 25.65 (95% CI 24.33–26.98)/million doses for ChAdOx1-S (Figure 1). The incidence rates of anaphylactic reactions were substantially lower as captured in the VAERS, with smaller differences among vaccines. The rates of anaphylactic reactions were approximately 1.6-fold (1.57, 95% CI 1.25–1.98, *p* < 0.001) higher for AD26.COV2, and ~4-fold higher for BNT162b2 (4.52, 95% CI 4.25–4.80, *p* = 0.000) or mRNA-1273 (4.11, 95% CI 3.78–4.47, *p* < 0.001), in EudraVigilance compared to the VAERS (Figure 1). The very low incidence of anaphylactic shock also varied by vaccine, particularly as captured in EudraVigilance, but differences were less pronounced compared to anaphylactic reactions. The maximum anaphylactic shock incidence identified was 5.21 (95% CI 4.61–5.81)/million doses for ChAdOx1-S.

### 3.3. Anaphylaxis Incidence Variation by Technological Platform of Vaccines

**Figure 2:**
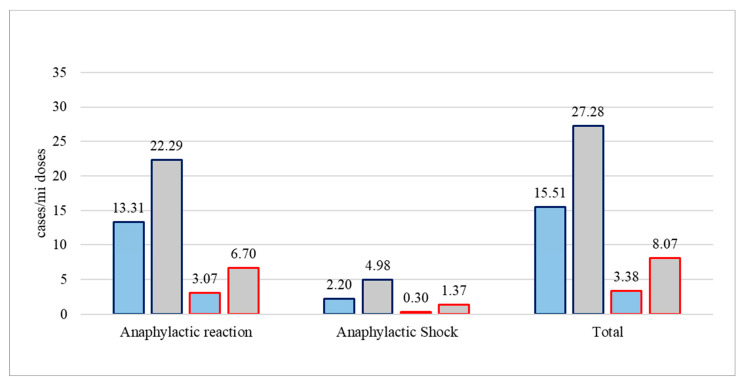
Rates of estimated anaphylactic reaction and anaphylactic shock post-COVID-19-vaccination for mRNA (blue bars) or vectored (grey bars) vaccines, as reported by the EudraVigilance (dark blue lining) and VAERS (red lining) databases, expressed per million administered doses from week 52, 2020 to week 2, 2023 for EudraVigilance and week 1, 2023 for VAERS.

Considering vaccine platforms, the incidence rates of anaphylactic reactions to vaccines based on adenovirus vectors were almost twofold higher compared to those of mRNA-based vaccines (22.29 (95% CI 21.2–23.38)/million vs. 13.31 (95% CI 13.04–13.59)/million vaccine doses in EudraVigilance, and 6.70 (95% CI 5.53–7.86)/million vs. 3.07 (95% CI 2.93–3.20)/million doses in the VAERS) (Figure 2). Anaphylactic shock incidence rates were also similarly higher for vectored compared to mRNA vaccines (4.98 (95% CI 4.47–5.50)/million vs. 2.20 (95% CI 2.08–2.31)/million doses in EudraVigilance, and 1.37 (95% CI 0.84–1.90)/million vs. 0.30 (95% CI 0.26–0.35)/million doses in the VAERS).

### 3.4. Anaphylaxis Incidence by Sex and Age

Detailed demographic data and outcomes of anaphylactic reaction and anaphylactic shock cases post-COVID-19-vaccination reported by EudraVigilance and the VAERS are presented in Table 1 and Table 2, respectively. The vast majority of these cases affected females (8407/10,439 + 1653/2110 = 10,060/12,549 or ~80% of all anaphylactic reaction and 1316/1817 + 170/222 = 1486/2039 or ~73% of all anaphylactic shock reports). With regard to age, different patterns are evident. In EudraVigilance, both types of anaphylaxis were more common among the working age population (18–64 years) and older individuals (65–85 years) than children and adolescents; meanwhile, in VAERS, more than half of both anaphylaxis types were experienced by subjects aged 30–59 years (1171/2110 = 55.5% of anaphylactic reactions and 123/222 = 55.4% of anaphylactic shock cases), and relatively high anaphylaxis incidence report rates in both younger (18–29 years) and older adult subjects (aged 65–79 years) in the VAERS are also noteworthy.

### 3.5. Outcome of Anaphylaxis Post-COVID-19-Vaccination

Regarding the outcome, the majority of post-COVID-19-vaccination anaphylactic reaction and anaphylactic shock cases with a known outcome (69.7% (7278/10,439) and 59.2% (1076/1817), respectively), were resolved or resolving, as captured by EudraVigilance (Table 1). The disease course was complicated in 17.4% (368/2110) of anaphylactic reaction and 27.5% (61/222) of anaphylactic shock cases, as captured in the VAERS (Table 2).

Fatalities were extremely rare, and mostly related to anaphylactic reaction incidents as opposed to anaphylactic shock post-COVID-19-vaccination (52 vs. 28 of 80 events in EudraVigilance and 10 vs. 3 of 13 events in the VAERS) (Table 1, Table 2, Table 3 and Table 4). The respective incidence rates of fatalities attributed to anaphylactic reaction and anaphylactic shock post-COVID-19-vaccination were 0.07 (95% CI 0.05–0.09)/million and 0.04 (95% CI 0.02–0.05)/million vaccine doses in Europe vs. 0.02 (95% CI 0.01–0.02)/million and 0 (95% CI 0–0.01)/million vaccine doses in the US, with overall rates across continents of 0.04 (95% CI 0.03–0.06)/million and 0.02 (95% CI 0.01–0.03)/million doses, respectively.

**Figure 3:**
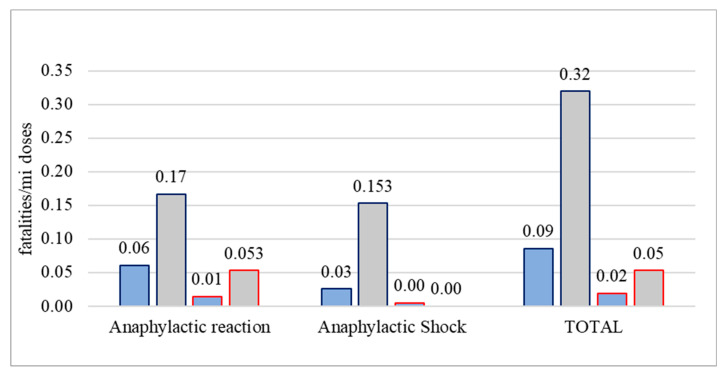
Rates of estimated anaphylactic reaction and anaphylactic shock fatalities post-COVID-19-vaccination for mRNA (blue bars) or vectored (grey bars) vaccines, as reported by EudraVigilance (dark blue lining) and the VAERS (red lining) databases, expressed per million administered doses from week 52, 2020 to week 2, 2023 for EudraVigilance and week 1, 2023 for the VAERS.

Considering the technological platform of vaccines, fatal outcomes occurred primarily post-vaccination with vectored rather than mRNA vaccines, with 3–6 times higher rates in Europe compared to the US (Figure 3). Individual deaths following adenovirus-based vectored vaccines were rare, but the number of administered doses of these vaccines was much smaller compared to mRNA vaccines (the denominator was smaller) (Table 1, Table 2, Table 3 and Table 4). For instance, regarding anaphylactic-reaction-associated deaths, 12/52 events in EudraVigilance and 1/10 events in the VAERS were recorded post-administration of vectored compared to mRNA vaccines, resulting in incidence rates of 0.17 (95% CI 0.07–0.26)/million vectored vaccine doses vs. 0.06 (95% CI 0.04–0.08)/million mRNA vaccine doses in Europe and 0.053 (95% CI −0.05–0.16)/million vectored vaccine doses vs. 0.01 (95% CI 0.00–0.02)/million mRNA vaccine doses in the US (Figure 3, Table 1 and Table 3). As for anaphylactic-shock-associated deaths, 11/28 events recorded in EudraVigilance were associated with vectored vaccines, resulting in incidence rates of 0.153 (95% CI 0.06–0.24)/million vectored vaccine doses vs. 0.03 (0.01–0.04)/million mRNA vaccine doses (Figure 3, Table 2 and Table 4). The three anaphylactic-shock-associated deaths reported in the VAERS occurred after vaccination with mRNA vaccines (3 events out of 644,019,542 administered vaccine doses).

## 4. Discussion

The incidence of anaphylactic reactions associated with the COVID-19 vaccination that we found during the two years of follow-up from late 2020 to early 2023 was slightly higher in Europe than in the US, and there was an overall reduction compared to the rates we assessed up to week 39 in 2021 (overall 8.96/million vaccine doses (14.19/million in the EEA and 3.17/million doses in the US) vs. overall 9.91/million vaccine doses (13.69/million in the EEA and 4.44/million doses in the US)) [12]. The updated incidence of anaphylactic shock associated with COVID-19 vaccination was slightly higher compared to the previously estimated rates, though US rates were slightly lower (1.46/million vaccine doses overall (2.47/million in the EEA and 0.33/million doses in the US) vs. 1.36/million vaccine doses overall (2.01/million in the EEA and 0.41/million doses in the US)) [12].

If true, it remains to be seen whether the observed decreases, particularly of anaphylactic shock post-vaccination, reflect improvements in the manufacturing of vaccines, underreporting of anaphylactic reactions to pharmacovigilance systems, or whether they possibly result from a better understanding and avoidance of the rare risks by susceptible individuals, according to issued guidelines for patients with “allergic history”. Whatever the answer, these results place the safety profile of COVID-19 vaccines within the range of reported anaphylactic reaction rates of other routine vaccines, between 1/million and 10/million vaccine doses [15]. Given the early-stage misconception about a presumably increased risk of anaphylaxis associated with these novel vaccines, especially of the mRNA platform, this evidence may offer further reassurance to the public regarding the safety of COVID-19 vaccines [11].

The incidence rates that we estimated for anaphylaxis related to mRNA vaccines in the US (2.94 (95% CI 2.77–3.11)/million for BNT162b2 and 3.26 (95% CI 3.04–3.49)/million doses for mRNA-1273) are slightly lower than previously reported population-based data (from 14 December 2020 to 26 June 2021) from the US Vaccine Safety Datalink, where researchers found rates of 4.8 (95% CI, 3.2–6.9) per million doses of BNT162b2 and 5.1 (95% CI, 3.3–7.6) per million doses of mRNA-1273 [16].

Our analysis further revealed differences in anaphylaxis rates, as captured in pharmacovigilance databases between Europe and the US, as well as between vaccines and vaccine platforms. In particular, we found higher incidence rates of anaphylaxis in EudraVigilance compared to the VAERS, both for the vector-based AD26.COV2.S and for the mRNA-based vaccines, which have stabilized to similar rates with regard to their association with anaphylaxis on each continent (13.41 (95% CI 12.78–14.05)/million mRNA-1273 vaccine doses vs. 13.29 (95% CI 12.98–13.60)/million BNT162b2 vaccine doses in EudraVigilance and 3.26 (95% CI 3.04–3.49)/million mRNA-1273 vaccine doses vs. 2.94 (95% CI 2.77–3.11)/million BNT162b2 vaccine doses in the VAERS). It is interesting that anaphylactic reactions following vaccination with AD26.COV2.S were lower compared to mRNA vaccines in Europe, but 2 times higher in the US.

Could these observations be related to the manufacturing and chemical composition of the two vaccine platforms? Although allergic reactions are well known and described in detail, the cause(s) that may trigger them after vaccination remains elusive [4]. These potential adverse effects may be attributed to various causes, which include: (i) the components of the final pharmaceutical product, i.e., the active ingredient (antigen) and excipients that are described in the summary of product characteristics of the European Public Assessment Report (EPAR) for each vaccine; (ii) the impurities or “related materials” that may be unintentionally present in the final formula; iii) the packaging material, in particular, the rubber stopper. Issues of gaps in reporting anaphylaxis rates should be considered, as well.

Regarding the components of the final pharmaceutical product, the spike glycoprotein of SARS-CoV-2 has been implicated in triggering allergic reactions possibly via the receptor-binding domain (RBD) binding to human ACE2B (437–508), RBD (319–541), and fusion peptides 1 (816–837) and 2 (835–855), which have been computationally predicted as allergenic regions [17]. However, the time lag needed by the cells for synthesis after vaccination for both vaccine platforms does not support this hypothesis. Anaphylactic reactions usually occur immediately or within a short time period after vaccination [4].

The molecule that has been widely suspected as the cause of anaphylactic reactions after the administration of the novel mRNA vaccines is polyethylene glycol 2000 (PEG 2000), a nonionic macromolecule that is conjugated to a phospholipid. This molecule renders the nanoparticle invisible from the reticuloendothelial system (RES), thereby avoiding opsonization and prolonging its half-life in blood circulation [18]. The anaphylactic reactions that were reported after vaccination with adenovirus vector vaccines were attributed to polysorbate 80 (TWEEN 80), which is used as a surfactant [4]. Both ingredients are widely used in many products, ranging from cosmetics and foods to drugs, either for topical or parenteral administration. Their extensive use will lead to the sensitization of the population to both molecules.

The development of cross-reactivity between PEG and polysorbates following exposure to these molecules has also been described [19]. This possible cross-reactivity was the reason behind the guidance issued by competent authorities worldwide, which recommended exclusion of persons with a known history of severe allergic reaction related to any vaccine components (especially PEG and polysorbates) [20,21]. Apart from the presence of PEGylated lipids in the lipid bilayers of liposomes, several physicochemical characteristics, such as their size, charge, and the molar ratio of cholesterol, may trigger a complement-activation-related pseudoallergy (CARPA), a non-IgE-mediated pseudoallergic reaction [22].

Regarding impurities or “related materials” of several other types that may be included in the formulas of vaccines, these components may be residuals of material used during DNA production (e.g., cell culture materials) or mRNA preparation and modification. Purification procedures are expected to remove most of such impurities (trace components), but this goal may not always be technically feasible. Impurities may also be created during mRNA vaccine storage, even if low temperatures are maintained. The stability of the mRNA molecule is ensured through pH regulation by using appropriate buffers or cryoprotectants [23]. Some buffer components (phosphates) and cryoprotectants (sucrose) are reported to crystallize at low temperatures, resulting in major pH changes, possibly leading to mRNA instability [4].

Regarding the packaging material, the material of the rubber stopper is an elastomer that usually consists of one or more polymers and fillers, curing agents, anti-degradants, and plasticizer, among other additives. Due to their close contact, several elastomeric matrix ingredients may migrate into the pharmaceutical product. This process is governed by the chemical affinity and diffusion characteristics of the pharmaceutical product and the chosen packaging material, under the storage, transportation, and handling conditions [24]. mRNA-1273 and BNT162b2 use chlorobutyl and synthetic bromobutyl rubber stoppers, respectively, while AD26.COV2.S uses chlorobutyl with a fluoropolymer-coated surface. The exact composition of the BNT162b2 vaccine’s rubber stopper has not been clarified [25,26,27,28].

Halobutyls such as chloro- or bromobutyls are frequently used for the closure of injectable pharmaceutical products as they provide an adequate gas or water barrier, protecting the product from oxidation and allowing a long-term shelf life. During the sterilization process, which may involve heat or radiation, halobutyls may become friable and brittle. Thus, their integrity and hermetic sealing might be compromised, allowing air into the vial. Some stopper particles may even contaminate the final product. Although their behavior under these conditions has been studied and described, information on the impact of deep freezing (−80 °C), which is required during storage or transportation, is scarce [24,29].

The anaphylactic reactions and anaphylactic shock cases reported by EudraVigilance compared to the VAERS indicated significant differences exist between reporting systems, vaccine platforms, and manufacturers. The reasons for this variability may be cumulative deviations created by slight variations during the manufacturing process, transportation, and storage conditions, which could affect the stability of vaccines. For example, variations in the type or concentration of impurities could arise due to differences in the conditions of antigen production at different production sites worldwide. In addition, alterations in the length of the deep-freezing time periods may affect the capping material of some vials, potentially compromising their sealing capacity. Conceivably, the reported variability may also reflect population differences in the degree of sensitization to the suspected ingredients prior to vaccination as well as differences in the prevalence of atopy, which has been linked to anaphylactic incidents post-vaccination [4,30].

Anaphylactic reactions were the main adverse effects of special interest reported mainly for mRNA vaccines at the launch of the global vaccination campaign in December 2020 [7]. As the number of vaccine doses increased, anaphylaxis associated with vector-based vaccines also appeared at rates comparable to or higher than (in the case of ChAdOx1-S) mRNA vaccines, even though their excipients differ significantly [11]. This fact may be attributed to the cross-reactivity between PEG 2000 and TWEEN 80 in the formulas of these vaccines. If true, should we anticipate increased anaphylaxis rates following first-time or booster vaccination with vaccines belonging to different platforms according to the so-called heterologous vaccination (mix-and-match) approach? This question demands further research and monitoring.

The risk of adverse events after vaccines, in general, including vaccination after COVID-19, is higher in women for reasons that are not fully understood. It is assumed that gender-specific factors including hormonal and genetic differences influence the immune response to the vaccines [31]. Furthermore, females are at increased risk of mast cell (MC)-related diseases, including anaphylaxis, and it is possible that gender differences in the MC phenotype are established in childhood [32].

Limitations of our work, intrinsic to passive pharmacovigilance reporting systems, should be discussed. The likely underreporting of anaphylaxis that generally holds for passive surveillance systems may be a potential limitation of the study, although COVID-19 vaccines have been under scrutiny since the beginning of their deployment. The unavailability of detailed information on vaccination (dose number, booster or heterologous vaccines) is another limitation of the study. Furthermore, potentially inaccurate mechanistic explanations may be introduced by the terminology used for the categorization of anaphylaxis post-vaccination in the two systems. In addition, other limitations, related to passive reporting systems, may stem from uncorrected errors in reporting (e.g., duplicate or incomplete records) and the fact that recorded events do not establish causality. However, the VAERS displayed a comparable detection sensitivity to other systems for important adverse events following vaccination, concerning anaphylaxis and Guillain–Barré syndrome [33]. Moreover, our analysis was based on real-life data from two of the world’s largest and most reliable pharmacovigilance databases. The good agreement of our estimated anaphylaxis incidence rates with those of population-based data further validates our findings and analysis.

## 5. Conclusions

Albeit rare, anaphylactic reactions were the most common allergic reactions after COVID-19 vaccination, as recorded in EudraVigilance and the VAERS during the study period. Their outcome was typically favorable, but their causes remain elusive. Our study revealed that differences exist in reported anaphylaxis rates, as captured in pharmacovigilance databases between Europe and the US, as well as between vaccines and vaccine platforms. Understanding the reasons behind true differences could lead to further optimization of the formulation of COVID-19 vaccines.

The use of any vaccine (or systemic medication) may be associated with a risk of developing severe allergic reactions; however, it is clear that the benefits of vaccination outweigh the potential risks, a statement that is particularly true for COVID-19. We found decreasing incidence rates of anaphylactic reactions during the two-year pandemic period of follow-up, and we found reactions were mostly associated with vectored rather than mRNA-based vaccines. Moreover, the obtained incidence rates were within the range of other commonly administered vaccines monitored by the same reporting systems. This finding provides additional reassurance to the public about the safety of COVID-19 vaccines. We hope our analysis will also help support efforts to further improve the safety of COVID-19 vaccines, which prevent severe disease.

## Figures and Tables

**Table 1:** Description of anaphylactic reaction cases post-COVID-19-vaccination reported by EudraVigilance from week 52, 2020 to week 2, 2023 *.

Vaccine		mRNA-1273				BNT162b2				ChAdOx1-S				AD26.COV2.S			
N **		128,739,510				534,676,385				56,019,836				**16,023,058**			
	**Sex**	**♀**	**♂**	NS	**Total**	**♀**	**♂**	NS	**Total**	**♀**	**♂**	NS	**Total**	**♀**	**♂**	NS	**Total**
**Age group**																	
NS		60	7	10	**77**	323	50	60	**433**	111	12	18	**141**	8	1	3	**12**
0–1 month		0	0	0	**0**	2	0	0	**2**	1	0	0	**1**	0	0	1	**1**
2 months–2 years		1	0	0	**1**	0	1	1	**2**	1	0	0	**1**	0	0	0	**0**
3–11 years		0	0	0	**0**	21	21	1	**43**	0	0	0	**0**	0	0	0	**0**
12–17 years		7	6	0	**13**	146	84	3	**233**	0	1	0	**1**	0	0	0	**0**
18–64 years		1128	324	13	**1465**	4756	795	81	**5632**	950	168	38	**1156**	74	66	9	**149**
65–85 years		125	37	0	**162**	473	149	9	**631**	100	28	4	**132**	4	3	0	**7**
>85 years		7	2	0	**9**	105	25	0	**130**	4	1	0	**5**	0	0	0	**0**
**Total**		**1328**	**376**	**23**	**1727**	**5826**	**1125**	**155**	**7106**	**1167**	**210**	**60**	**1437**	**86**	**70**	**13**	**169**
**Outcome**		**Number of individual cases**															
Fatal		3				37				9				3			
Not recovered/Not resolved		176				459				148				15			
Recovered/Resolved		781				3903				603				82			
Recovered/Resolved with sequelae		19				93				28				1			
Recovering/Resolving		209				1405				279				16			
Unknown		539				1209				370				52			

NS, not specified. * Data (up to 14 January 2023) were summarized from the European Medicines Agency “EudraVigilance” European database of suspected adverse drug reaction reports [8]. ** The Total number of COVID-19 vaccine doses administered in Europe was 971,865,122 as of 17 January 2023. The distribution of vaccine doses by manufacturer was as follows: Moderna 128,739,510, Moderna Bivalent 864,538, Pfizer-BioNTech 534,676,385, Pfizer-BioNTech Bivalent 26,845,244, AstraZeneca 56,019,836, Johnson & Johnson 16,023,058, and other/unknown 208,696,551 [13].

**Table 2:** Description of anaphylactic shock cases post-COVID-19-vaccination reported by EudraVigilance from week 52, 2020 to week 2, 2023 *.

Vaccine		mRNA-1273				BNT162b2				ChAdOx1-S				AD26.COV2.S			
N **		128,739,510				534,676,385				56,019,836				**16,023,058**			
	**Sex**	**♀**	**♂**	NS	**Total**	**♀**	**♂**	NS	**Total**	**♀**	**♂**	NS	**Total**	**♀**	**♂**	NS	**Total**
**Age group**																	
NS		8	1	1	**10**	64	16	9	**89**	26	1	3	**30**	4	1	3	**8**
0–1 month		0	0	0	**0**	1	0	0	**1**	0	0	0	**0**	0	0	0	**0**
2 months–2 years		0	0	0	**0**	0	0	0	**0**	0	0	0	**0**	0	0	0	**0**
3–11 years		0	0	0	**0**	5	2	0	**7**	0	0	0	**0**	0	0	0	**0**
12–17 years		2	2	0	**4**	39	27	2	**68**	0	0	0	**0**	0	0	0	**0**
18–64 years		143	48	7	**198**	663	199	41	**903**	181	44	8	**233**	32	16	9	**57**
65–85 years		23	9	1	**33**	82	31	7	**120**	23	6	0	**29**	1	1	0	**2**
>85 years		3	1	0	**4**	16	5	0	**21**	0	0	0	**0**	0	0	0	**0**
**Total**		**179**	**61**	**9**	**249**	**870**	**280**	**59**	**1209**	**230**	**51**	**11**	**292**	**37**	**18**	**12**	**67**
**Outcome**		**Number of individual cases**															
Fatal		6				11				9				2			
Not recovered/Not resolved		31				102				27				11			
Recovered/Resolved		105				588				104				18			
Recovered/Resolved with sequelae		8				53				12				0			
Recovering/Resolving		33				165				54				9			
Unknown		66				290				86				27			

NS, not specified. * Data (up to 14 January 2023) were summarized from the European Medicines Agency “EudraVigilance” European database of suspected adverse drug reaction reports [8]. ** The Total number of COVID-19 vaccine doses administered in Europe was 971,865,122 as of 17 January 2023. The distribution of vaccine doses by manufacturer was as follows: Moderna 128,739,510, Moderna Bivalent 864,538, Pfizer-BioNTech 534,676,385, Pfizer-BioNTech Bivalent 26,845,244, AstraZeneca 56,019,836, Johnson & Johnson 16,023,058, and other/unknown 208,696,551 [13].

**Table 3:** Description of anaphylactic reaction cases post-COVID-19-vaccination reported by the VAERS from week 52, 2020 to week 1, 2023.

Vaccine		mRNA-1273				BNT162b2				AD26.COV2.S			
N *		249,495,031				397,196,666				18,956,612			
	**Sex**	**♀**	**♂**	NS	**Total**	**♀**	**♂**	NS	**Total**	**♀**	**♂**	NS	**Total**
**Age group**													
Unknown		49	8	22	**79**	99	16	51	**166**	11	1	16	**28**
6–11 months		1	1	0	**2**	0	0	0	**0**	0	0	0	**0**
3–5 years		0	1	0	**1**	0	0	0	**0**	0	0	0	**0**
6–17 years		0	1	0	**1**	38	24	1	**63**	0	0	0	**0**
18–29 years		80	19	0	**99**	111	23	1	**135**	9	7	0	**16**
30–39 years		133	30	2	**165**	156	41	0	**197**	20	5	0	**25**
40–49 years		144	15	2	**161**	195	29	1	**225**	19	6	0	**25**
50–59 years		135	23	0	**158**	178	22	0	**200**	11	4	0	**15**
60–64 years		42	10	0	**52**	59	15	0	**74**	7	1	0	**8**
65–79 years		61	25	1	**87**	72	21	2	**95**	7	2	0	**9**
>80 years		6	3	0	**9**	9	5	0	**14**	1		0	**1**
**Total**		**651**	**136**	**27**	**814**	**917**	**196**	**56**	**1169**	**85**	**26**	**16**	**127**
Outcome **		**Number of individual cases**											
Fatal		2	0	1	3	2	4	0	6	1	0	0	1
Complicated course		110	28	2	140	161	31	4	196	18	12	2	32

NS, not specified. * The Total number of COVID-19 vaccine doses administered in the US was 716,372,081 as of 11 January 2023, with the following distribution by manufacturer: Moderna 249,495,031, Moderna Bivalent 18,042,918, Pfizer-BioNTech 397,196,666, Pfizer-BioNTech Bivalent 31,817,560, Johnson & Johnson 18,956,612, and Novavax 71,979 [14]. ** Only severe outcome categories were included for clarity. Data (until 6 January 2023) for complete entries were summarized from the Vaccine Adverse Event Reporting System (VAERS) [9].

**Table 4:** Description of anaphylactic shock cases post-COVID-19-vaccination reported by the VAERS from week 52, 2020 to week 1, 2023.

Vaccine		mRNA-1273				BNT162b2				AD26.COV2.S			
N *		249,495,031				397,196,666				18,956,612			
	**Sex**	**♀**	**♂**	NS	**Total**	**♀**	**♂**	NS	**Total**	**♀**	**♂**	NS	**Total**
**Age group**													
Unknown		9	1	3	**13**	11	4	1	**16**	2	0	3	**5**
6–11 months		0	0	0	**0**	0	0	0	**0**	0	0	0	**0**
3–5 years		0	0	0	**0**	0	0	0	**0**	0	0	0	**0**
6–17 years		0	0	0	**0**	2	2	0	**4**	1	0	0	**1**
18–29 years		7	7	0	**14**	7	1	0	**8**	2	1	0	**3**
30–39 years		8	1	0	**9**	16	1	0	**17**	5	0	0	**5**
40–49 years		18	5	1	**24**	20	4	0	**24**	5	1	0	**6**
50–59 years		11	5	0	**16**	14	4	0	**18**	4	0	0	**4**
60–64 years		3	0	0	**3**	6	0	0	**6**	1	0	0	**1**
65–79 years		8	4	0	**12**	6	2	0	**8**	1	0	0	**1**
>80 years		1	1	0	**2**	2	0	0	**2**	0	0	0	**0**
**Total**		**65**	**24**	**4**	**93**	**84**	**18**	**1**	**103**	**21**	**2**	**3**	**26**
Outcome **		**Number of individual cases**											
Fatal		1	0	0	1	2	0	0	2	0	0	0	0
Complicated course		17	7	0	24	26	5	0	31	6	0	0	6

NS, not specified. * The total number of COVID-19 vaccine doses administered in the US was 716,372,081 as of 11 January 2023, with the following distribution by manufacturer: Moderna 249,495,031, Moderna Bivalent 18,042,918, Pfizer-BioNTech 397,196,666, Pfizer-BioNTech Bivalent 31,817,560, Johnson & Johnson 18,956,612, and Novavax 71,979 [14]. ** Only severe outcome categories were included for clarity. Data (until 6 January 2023) for complete entries were summarized from the Vaccine Adverse Event Reporting System (VAERS) [9].

## Data Availability

Two publicly available datasets were analyzed in this study. The data can be found here: https://www.adrreports.eu (accessed on 13 January 2023) and https://vaers.hhs.gov/about.html (accessed on 6 January 2023).
